# Clinicopathological and Molecular Characteristics of Colorectal Signet Ring Cell Carcinoma: A Review

**DOI:** 10.3389/pore.2021.1609859

**Published:** 2021-07-26

**Authors:** Yang An, Jiaolin Zhou, Guole Lin, Huanwen Wu, Lin Cong, Yunhao Li, Xiaoyuan Qiu, Weikun Shi

**Affiliations:** ^1^ Department of General Surgery, Peking Union Medical College Hospital, Chinese Academy of Medical Sciences and Peking Union Medical College, Beijing, China; ^2^ Department of Pathology, Peking Union Medical College Hospital, Chinese Academy of Medical Sciences and Peking Union Medical College, Beijing, China

**Keywords:** colorectal cancer, clinicopathology, signet ring cell carcinoma, molecular features, review

## Abstract

Colorectal signet ring cell carcinoma (SRCC) is a rare subtype of colorectal cancer (CRC) with unique characteristics. Due to the limited researches on it, a comprehensive and in-depth understanding of this subtype is still lacking. In this article, we summarize the clinicopathological features and molecular characteristics of colorectal SRCC based on a literature review. Clinically, SRCC has been associated with young age, proximal site preference, advanced tumor stage, high histological grade, high rate of lymph node involvement, frequent peritoneal metastasis, and a significantly poor prognosis. Regarding molecular characteristics, in SRCC, the mutation burden of the classic signaling pathways that include WNT/β-catenin, RAS/RAF/MAPK, and PI3K/AKT/mTOR signaling pathways are generally reduced. In contrast, some genes related to the “epithelial-mesenchymal transition (EMT) process” and the “stem cell properties”, including *RNF43*, *CDH1*, and *SMAD4*, as well as the related TGF-β signaling pathway have been observed more frequently altered in SRCC than in conventional adenocarcinoma (AC). In many studies but not in others, SRCC showed a higher frequency of BRAF mutation, microsatellite instability-high (MSI-H) and CpG island methylator phenotype (CIMP) positive status compared to AC. It has been proposed that colorectal SRCC consists of two subtypes, in which the MSI^+^/CIMP^+^/*BRAF* ^+^/CD3^+^/PD-L1^+^ hypermethylated genotype is more common in the proximal colon, and may represent the potential candidate for immunotherapy. Understanding the special molecular mechanisms related to the aggressive biology of SRCC is of great importance, which may provide a theoretical basis for the development of more targeted and effective treatments for this refractory disease.

## Introduction

Colorectal cancer (CRC) ranks the third most common cancer and the second leading cause of cancer-related death globally [1,2]. First proposed by Saphir and Laufman in 1951 [3], signet ring cell carcinoma (SRCC) is a rare subtype of CRC, by definition composed of at least 50% of neoplastic cells showing signet ring cell (SRC) morphology. Among all subtypes of CRC, the conventional adenocarcinoma (AC) accounts for the vast majority, the mucinous adenocarcinoma (MAC) accounts for 10–15%, and SRCC only accounts for ∼1% [4–8]. During the past 3 decades, epidemiology data of the United States showed an overall decline in the incidence of CRC, while a rising trend has been observed for young adult patients [9–11]. Several studies have shown a more aggressive manifestation for the early-onset CRC and an increased incidence of SRC histology [9,12]. Because of the rarity of colorectal SRCC, many aspects of it have not been fully elucidated. In this article, we describe and discuss the clinicopathological features and molecular characteristics of colorectal SRCC based on a literature review. We searched MEDLINE and PreMEDLINE database for English-language articles and references from relevant articles. Search terms included “signet ring cell”, “colorectal”, “colon”, “rectum”, “carcinoma”, “cancer”, “epidemiology”, “clinicopathological”, “molecular”, “genotype”, “mutation”, “microsatellite instability”, “BRAF”, “prognosis”, “survival”, “metastasis”, “surgery”, “chemotherapy”, and “treatment”. We summarized the results of studies that analyzed the clinicopathological and/or molecular characteristics of colorectal SRCC based on population-based registries, single-center or multi-center cohorts, published between January 1999 and January 2021. Studies that did not distinguish mucinous adenocarcinoma from SRCC were ruled out. Articles solely reported in the form of abstracts or meeting reports are excluded.

## Clinicopathological Features of Colorectal Signet Ring Cell Carcinoma

**FIGURE 1:**
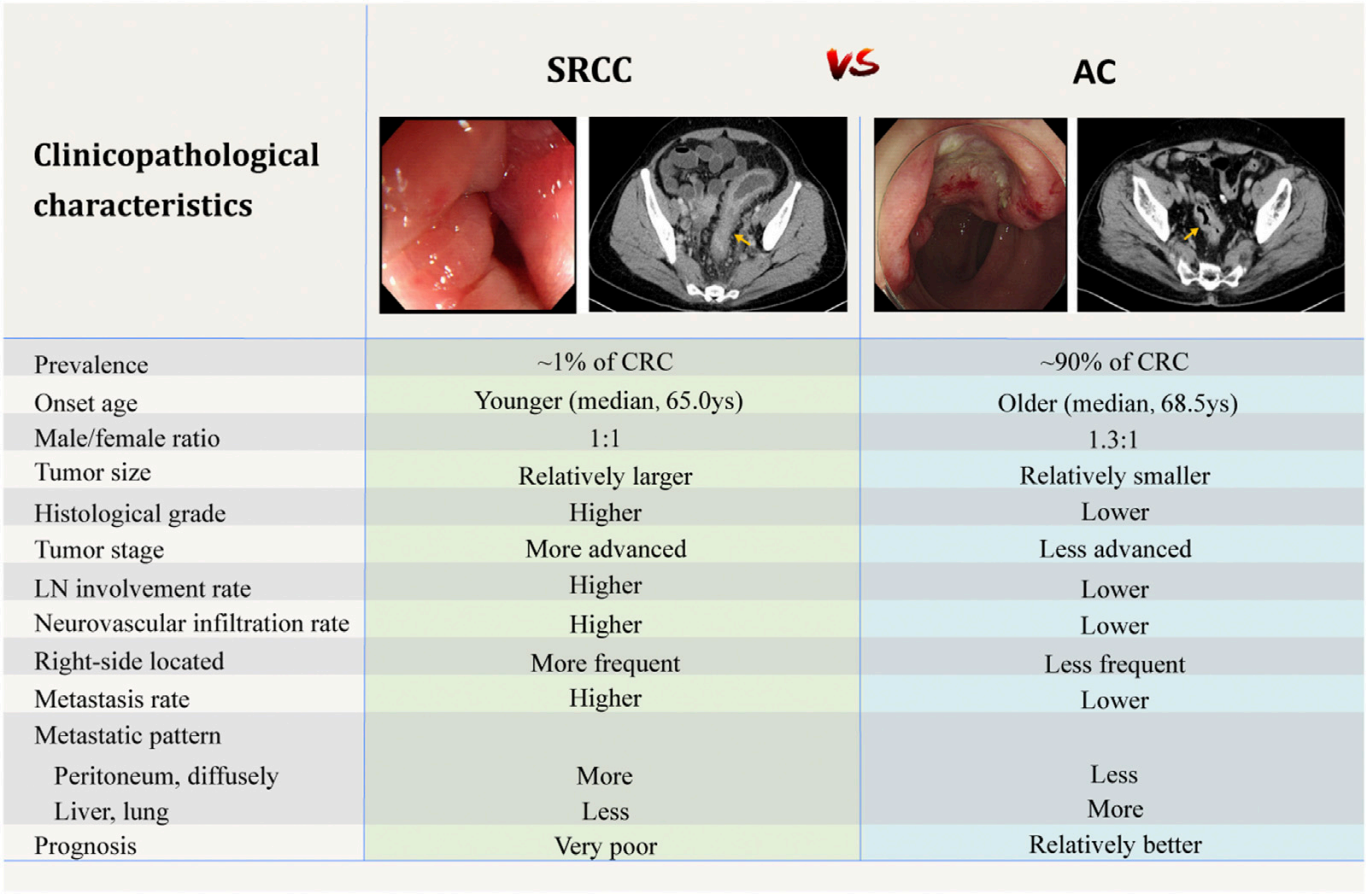
Comparison of clinicopathological characteristics between SRCC and AC [4–6,20,91]. The above endoscopic and CT images are selected by the authors from the clinical database of Peking Union Medical College Hospital. Related patients have provided written informed consents for publication of their clinical data. Adobe Illustrator CC 2018 was used to create the artwork.

Being a kind of poorly cohesive carcinoma [13], colorectal SRCC is a distinct entity with different clinical manifestations, pathological features, and biological behaviors compared to AC. Studies upon the clinicopathological features of colorectal SRCC were summarized in Table 1 and Figure 1.

**TABLE 1:** Clinicopathological features of colorectal signet ring cell carcinoma.

Study	Year	Study type	No. of SRCC (%)	Age (years)	Gender M/F	Location (%)			High grade (G3/4)	TNM stage III–IV	Stage N+ (LN positive) (%)	Angio-invasion	Site of metastasis (%)							Prognosis	
						Proximal colon	Distal colon	Rectum					Peritoneum	Ovary	Liver	Lung	Bone	Distant LN	Others	Survival rate (%)	Survival (months)
Psathakis, 1999 [24]	1979–1997	Retro. Single-center (Germany)	14 (0.88%)	67.5 ± 16.9	1.0:1	50.0%	28.6%	21.4%	—	92.9%	—	—	64.3%	7.1%	14.3%	0	0	—	14.3%	3-year OS, 0.0%	Median OS, 14 ms
^†^Kang, 2005 [16]	1991–2000	Review of SEER data	1,522 (0.9%)	65.9 ± 16.6	1.0:1	60.0%	18.6%	21.4%	73.5%	80.9%	—	—	—	—	—	—	—	—	—	5-y RS	—
																				26.8%	
Sung, 2008 [27]	1995–2006	Retro. Single-center (Korea)	65 (NA)	50.8 ± 17.2	1.7:1	35%	65% (left-sided)		—	89%	pN+, 88%; pN2, 77%	89%	—	—	—	—	—	—	—	3-year Cumulative OS, 33%	Mean OS, 48.4 ms

Chew, 2010 [90]	1999–2005	Retro. Single-center (Singapore)	30 (1.1%)	63.5 (median)	0.4:1	27%	46%	27%	77%	94%	89%	—	50%		7%	22%	—	—	—	5-year CSS, 11.1%	—
Mizushima, 2010 [38]	1993–2007	Review of Osaka database (Japan)	19 (0.32%)	65.5 ± 10.9	0.7:1	55.6%	5.6%	38.9%	—	—	pN+, 73.7%; pN2, 47.4%	—	31.6%	—	10.5%	0	0	5.3%	—	5-year OS, 24.1%	Median OS, 15 ms
Hyngstrom, 2012 [6]	1998–2002	Review of NCDB data	2,260 (1%)	18–49 years, 19%; 50–75 years, 51%; 76–90 years, 29%	1.0:1	62%	19%	20%	77%	80%	—	—	—	—	—	—	—	—	—	—	—
Kakar, 2012 [28]	-	Retro. Multi-center (United States)	33 (NA)	56.4 (mean)	2.7:1	48%	52% (left-sided)		—	79%	—	—	—	—	—	—	—	—	—	—	—
Nitsche, 2013 [8]	1982–2012	Retro. Single-center (Germany)	30 (0.9%)	64 (median)	1.7:1	50%	13%	37%	90%	87%	pN+, 83%	17%	—	—	—	—	—	—	—	5-years CSS, 21 ± 8%	Median CSS, 10 ms
											pN2, 73%										
Thota, 2014 [29]	1995–2009	Review of VACCR data	206 (0.6%)	67 (median)	33.3:1*	75.6%	24.4%	NA	85.5%	78.8%	pN+, 69.1%	—	—	—	—	—	—	—	—	5-years OS of Stage III, 19%	Median OS, 18.6 ms
											pN2, 44.6%										
Hugen, 2015 [5]	1989–2010	Review of NCR data (Dutch)	1,972 (1%)	70 (median)	1.0:1	59.7%	22.3%	18.0%	—	78.0%	—	—	—	—	—	—	—	—	—	Colon: 5-y RS, 30.8%	—
																				Rectum: 5-y RS, 19.5%	
Nitsche, 2016 [14]	1998–2012	Review of Munich Cancer Registry (Germany)	160 (0.6%)	66 ± 15	1.2:1	77.8%	9.2%	13.1%	96.2%	85.7%	pN+, 70.5%	38.0%	—	—	—	—	—	—	—	5-year OS, 40.3%	-—
											pN2, 47.7%										
Liang, 2017 [91]	1990–2010	Retro. Single-center (China)	37 (1.4%)	50 (median)	1.5:1	48.6%	5.4%	45.9%	—	89.1%	pN+, 70.3%	—	66.7%	—	19.1%	4.8%	4.8%	-	9.6%	5-year OS, 10.8%	Mean OS, 27.1 ± 3.3 ms
											pN2, 51.2%										
Korphaisarn, 2019 [20]	2009–2015	Retro.	35^#^	55 (median)	0.9:1	62.9%	37.1% (left-sided)		100%	100%	—	—	82.9%	—	17.1%	17.1%	—	—	—	—	Median OS, 16.4 ms
		Single-center																			
		+UTMDACC registry																			
^†^Shi, 2019 [4]	2010–2014	Review of SEER data	1,932 (1.11%)	<65, 48.24%	1.1:1	—	—	—	93.14%	77.93%	pN+ 64.38%	—	17.65%	—	6.88%	2.80%	3.05%	—	11.60%	—	(PM cases)
																					Median OS, 9 ms; Median CSS, 10 ms
^†^Benesch, 2020 [7]	1975–2016	Review of SEER data	4,586 (1.0%)	65.2 ± 16.4	1.0:1	Only colon SRCC included		—	93.0%	—	—	—	—	—	—	—	—	—	—	5-year OS, 33.6%	Median OS, 21.6 ms
																				10-year OS, 28.6%	
^†^Yang, 2020 [30]	2004–2015	Review of SEER data	3,278 (NA)	63 (median)	1.0:1	63.19%	16.41%	20.40%	93.46%	81.27%	pN+, 73.03%	—	—	—	—	—	—	—	—	5-year OS, 25.14%	Median OS, 16.0 ms
											pN2, 70.41%									5-year CSS, 29.32%.	

SRCC, signet ring cell carcinoma; M, male; F, female; LN, lymph node; Retro., retrospective; OS, overall survival; RS, Relative Survival; NA, not available; CSS, cancer-specific survival; *, There is gender bias in this database; ^#^, all patients were with stage IV tumors; PM, peritoneal metastasis. ^†^, The four articles are all based on SEER database with overlapping time frames. The included patients could be partially duplicated. Due to differences in inclusion/exclusion criteria, analysis parameters, study methods, and time spans, all the four studies are included.

## Patient Demographics

SRCC are reported to be diagnosed at younger age compared to AC [5,6,9,14–16]. The age limit defining early-onset CRC varies from studies to studies, while under 40 years of age has been adopted by most authors [9,12,17–20]. Population-based studies using the surveillance, epidemiology, and end results (SEER) program cancer registry revealed a mean age at onset of about 65 years old for colorectal SRCC (Table 1), which is 3.5 years earlier than that of AC [7]. Although overall CRC is more common in men than in women, while according to large-scale studies, the incidence of colorectal SRCC in both sexes is roughly equal [5–7,21].

## Clinical Characteristics

Clinically, colorectal SRCC has some different characteristics from AC. Patients often have larger tumors and more advanced tumor stages at initial diagnosis [5,9,22]. Unlike the AC morphology of intraluminal mass, colorectal SRCC often appears as diffuse circumferential thickening of the bowel wall with markedly narrowed lumen, and sometimes presents similar to inflammatory disease [17,23]. Psathakis and others [24] analyzed patients’ primary symptoms at diagnosis and suggested that the frequently advanced stages may result from a delay in diagnosis, which may be attributed to the special features of colorectal SRCC including younger age at onset, atypical and delayed clinical manifestations, and the high false-negative rates of endoscopic biopsy [17,23]. Psathakis proposed that the characteristic long-term intramural tumor growth without penetrating the mucosa might be one explanation for the special features of SRCC [24].

## Distribution of Sites

It has been suggested that right- and left-sided CRC may arise by different mechanisms [25,26]. Although with some exceptions in reports of Asian population [27,28], most large-scale studies from western countries revealed a proximal colon (including the cecum, ascending and transverse colon) dominance for colorectal SRCC [5,6,14,16,29,30]. Overall, rectal cancer accounts for nearly 30% of all CRC [5,6,16,21,31]. In contrast, SRCC of the rectum is less common and accounts for ∼20% of all colorectal SRCC [5,6,16,30]. The embryological and genetic differences between the proximal colon that originates from the midgut, and the distal colon and rectum from the hindgut might partly explain the peculiarity of SRCC distribution.

## Aggressive Behavior

Colorectal SRCC has been identified as a subtype with aggressive biological behavior. In comparison with AC, SRCC usually has higher tumor grade and is diagnosed in more advanced stage [6,8,9,14,16,29] (Table 1). In addition, adverse histological features including higher percentage of locoregional lymph node involvement, lympho-vascular invasion and perineural infiltration are more common in colorectal SRCC [8,9,14,18,27] (Table 1).

Compared to AC patients, SRCC patients more frequently have local and distant metastasis and are more likely to have multiple-site tumor spread, which is characterized by a significantly higher incidence of peritoneal dissemination (more than 50%, as reported by the large-scale autopsy study of Hugen et al. [32]) and distant lymph node metastasis, and a lower incidence of hepatic and lung metastasis. In addition, SRCC patients show divergent metastatic pattern with involvement of rare metastatic sites including bone, brain, bone marrow, ovaries, skin, heart, and can present as multiple polypoid colonic lesions [8,14,15,32–34]. The underlying mechanism for this distinct metastatic pattern is unclear. Histologically, SRCs are usually present as single cells or in loose clusters. Some authors suggested that this may imply a lack of cell-cell adhesion, that is, the SRCs can loosen contact with surrounding structures, causing them to easily spread and form diffuse disseminations instead of forming larger metastatic foci [15,27,33,35].

## Prognosis

Colorectal SRCC has been associated with significantly worse prognosis than AC in terms of higher local and distant recurrence rate, shorter cancer-specific survival (CSS) and overall survival (OS) [5,7,8,14,16,24,36]. Most studies describe an over 50% increased hazard ratio (HR) of death from cancer compared to AC [5,6,14,16]. Prognostic factors that strongly correlated with the poor outcomes have been widely discussed. Among them, advanced stage at diagnosis is consistently believed the main reason for the poor prognosis. However, the prognostic value of SRC histology remains controversial. After adjustment for covariates including tumor stage and location, SRC histology has been shown an independent adverse prognostic factor in most studies [5,6,8,16,20,36,37]. While there are still a few authors who believe that the poor outcome only comes from the advanced stage at diagnosis and question the role of tumor biology in it [14,22]. Mizushima et al. [38] reported that patients with stage III colorectal SRCC had significantly worse survival than those with AC, while no such difference was observed in stage II or IV tumors. A population-based study including 1,972 colorectal SRCC cases demonstrated that SRCC was associated with significantly worse 5-year relative survival than AC. This survival difference was found in both stage II and III cases, but was most prominent in stage III [5].

Additionally, the high rate of synchronous and metachronous distant metastasis associated with the histological subtype has been supposed an important reason for the bleak prognosis of colorectal SRCC [5,8,39,40]. For patients with diffuse distant metastasis (most common as peritoneal dissemination), their disease can neither be radically removed by surgery, nor can it be effectively controlled by chemotherapy [41], which lead to the dismal outcomes of patients.

## Molecular Characteristics of Signet Ring Cell Carcinoma

**FIGURE 2:**
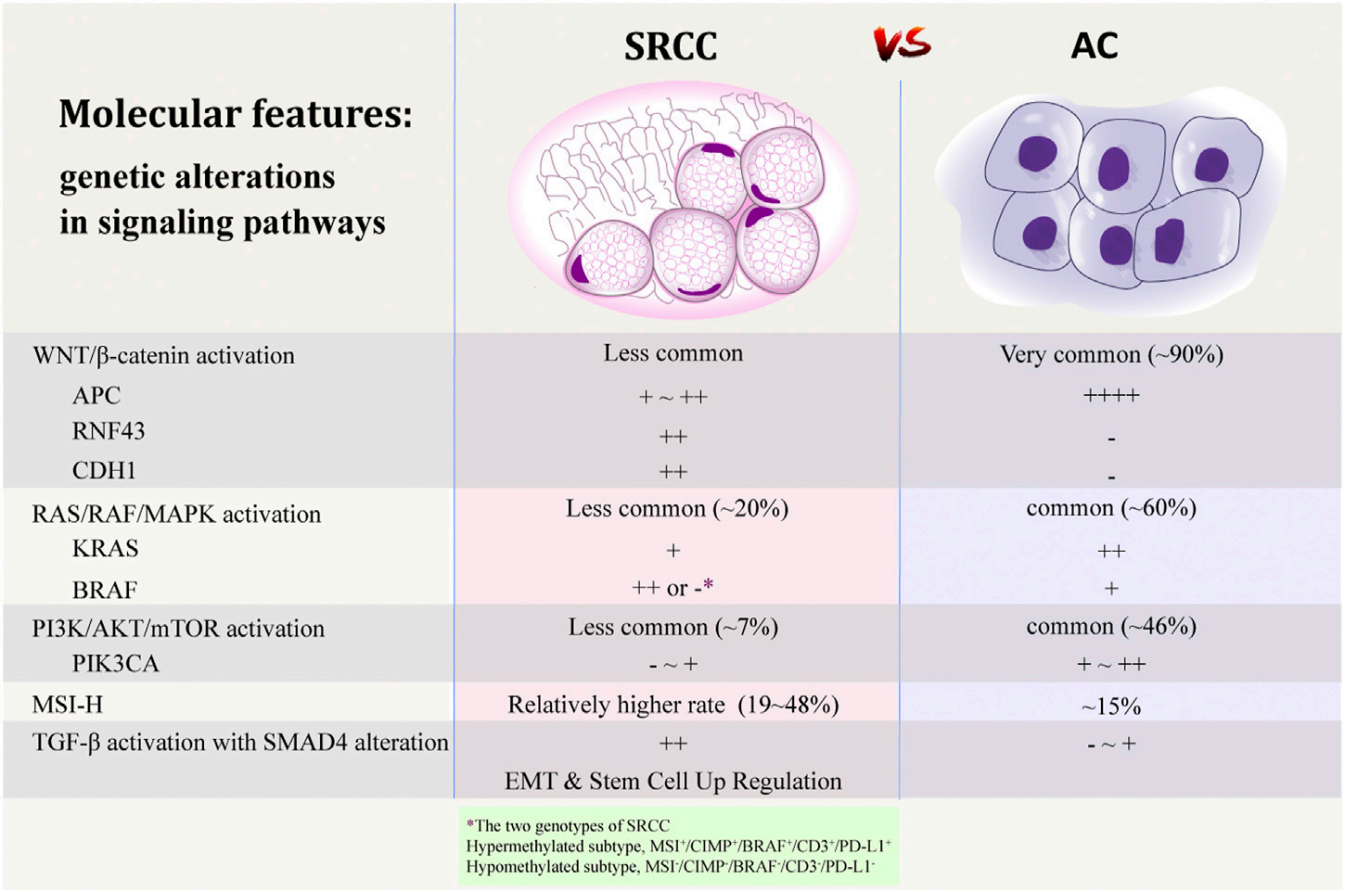
Comparison of molecular features between SRCC and AC [20,44,45,58–60,72,75,93]. Adobe illustrator CC 2018 was used to create the artwork.

In addition to having special clinicopathological characteristics, colorectal SRCC is also different from AC for the molecular features. Some authors suggested that SRCs may arise from a separate genetic pathway [35]. Due to the rarity of this subtype, both the number and sample size of studies focusing on its molecular abnormalities are very small. Although a thorough understanding and consensus has not yet been formed, some studies have analyzed and summarized the genetic, epigenetic, and protein expression characteristics of colorectal SRCC, which may contribute to the understanding of the carcinogenesis mechanism of SRCC, and may explain the unique clinicopathological characteristics and the poor prognosis of this particular subtype. A summary of previous studies upon the molecular features of colorectal SRCC is shown in Table 2 and Figure 2.

**TABLE 2:** Molecular features of colorectal signet ring cell carcinoma.

Study	Country	Sequencing assay	No.	Stage	SRC component (%)	Site, P:D	KRAS (%)	NRAS (%)	BRAF (%)	PIK3CA (%)	APC (%)	TP53 (%)	SMAD4 (%)	RNF43 (%)	KIT (%)	CDH1 (%)	MSI-H (%)	MSI-H tumor site, P:D	dMMR (IHC) (%)	CIMP positive (%)	p16 loss (%)	MLH1 loss (%)	LOH positive (%)
Kawabata, 1999 [92]	Japan	PCR-RFLP	10	II–IV	NA	0.25:1	11					29% (IHC)					30	2.0:1					
Kakar, 2005 [75]	United States	PCR	72	I–IV	>50%	1.26:1											31	4.3:1	29			29% (IHC)	
Ogino, 2006 [60]	United States	WGA-PCR	39	NA	Any	NA	26		28			50% (IHC)					31	—			29% (IHC)	30% (IHC)	18q LOH, 38%
Sung, 2008 [27]	Korea	PCR	63	II–IV	>50%	0.55:1											19	2.0:1					
Kakar, 2012 [28]	United States	PCR	33	I–IV	>50%	0.94:1	53		33								24	3.0:1		48			Any of the 4 loci, 93%; 18q LOH, 40%
Hartman, 2013 [76]	United States	PCR	53	I–IV	>50%	1.79:1			30								43	3.6:1				35.8% (IHC)	
Inamura, 2015 [61]	United States	PCR Pyrosequencing	17	I–IV	>50%	2.80:1	5.9		35	6.3							29	—		29		29%	
Wei, 2016 [58]	China	NGS	61	I–IV	Any	0.69:1	16.7	5.4	3.7		31.5		40.7			24.1						16.7%	
Alvi, 2017 [62]	Northern Ireland	NGS, Sanger seq.	44	I–IV	>50%	1.59:1	12		31	4	35	69%			34		48	9.0:1		41			
Yalcin, 2017 [77]	Turkey	PCR-RFLP Sanger seq.	28	II–IV	Any	0.87:1			39.3														
Nam, 2018 [45]	Korea	WES RNA seq.	5	II–IV	>50%	0.25:1	40	0	0	0	20	40%	20										
Kim, 2019 [59]	Korea	Targeted panel NGS	17	I–III	Any	0.55:1	23.5		5.9	5.9	23.5	47.1%	29.4				0	—					
Korphaisarn, 2019 [20]	United States	NGS	35	IV	≥50%	1.70:1	11.4	0	8.6	2.9	2.9	60.0%	14.3				12.1	—	12.1	33.3			
Li, 2020 [44]	China	WES	29	II–IV	>70%	0.26:1	10.3		6.9	0	3.4	55.2%	20.7	34.5			3.4	—					
Chen, 2020 [93]	China	NGS	18	I–IV	Any	1.57:1	11.1	11.1	5.6	22.2	27.8	55.6%	11.1		0				0				

SRC, signet ring cell; P:D, proximal colon: distal colon and rectum; MSI-H, microsatellite instability-high; dMMR, deficient mismatch repair; IHC, immunohistochemistry; CIMP, CpG island methylator phenotype; LOH, loss of heterozygosity; PCR, polymerase chain-reaction; RFLP, restriction-fragment length polymorphism; NA, not available; WGA, whole genome amplification; NGS, next generation sequencing; WES, whole exome sequencing; seq., sequencing.

## Adenomatous polyposis coli Mutation and WNT/β-Catenin Signaling Pathway

Adenomatous polyposis coli (*APC*) is a key tumor suppressor gene, which plays a critical early role in the tumorigenesis of most CRC [42]. Aberrant inactivation of *APC* results in activation of the WNT/β-catenin signaling pathway, which is a common event in the colorectal adenoma-carcinoma sequence [18]. Genomic analyses have shown that WNT pathway is altered in 93% of all CRC, including biallelic inactivation of *APC* or activating mutations of *CTNNB1* in ∼80% of cases [43]. However, colorectal SRCC is strikingly different from AC in terms of the mutated genes in the WNT pathway, showing significantly lower frequency of *APC* mutation [20,43–45].

Being a critical component of WNT pathway, β-catenin shows inappropriate stabilization and translocation to the nucleus when the signaling pathway is upregulated, which is considered a biomarker of WNT pathway activation [46–48]. Apart from *APC*, other genes in the WNT pathway were also found significantly less mutated in colorectal SRCC than those in AC, which included β-catenin target genes *LGR5*, *SOX9*, *AXIN2*, and *MSI1* [44]. However, study of Börger et al. using immunohistochemical analysis observed that the membranous localization of β-catenin was reduced and its nuclear expression was present in colorectal SRCC [35], which showed that the WNT/β-catenin signaling pathway may also be activated in SRCC. It is therefore proposed that the WNT pathway may be activated through alternative pathways in SRCC, in the absence of *APC* mutation. *RNF43*, another key regulator of WNT pathway, encodes transmembrane molecules that attenuate WNT signaling [48]. Li et al. observed that SRCC was associated with frequent mutations in *RNF43*, with nonsense mutations (p.Glu43* and p. Arg132*) enriched at the N-terminus, regardless of mutation burden. They proposed that SRCC may prefer a complete inactivation of *RNF43* to activate WNT/β-catenin pathway, instead of through *APC* mutation [44]. *CDH1*, encoding the cell adhesion protein E-cadherin, is commonly mutated in gastric SRCC. Studies have shown that *CDH1* loss activates WNT signaling by unleashing membrane-bound β-catenin, which in turn activates the WNT/β-catenin pathway [47,49–51]. In colorectal SRCC, *CDH1* mutation occurs in a proportion of cases and is found to be associated with reduction in E-cadherin in these tumors [52]. *CDH1* mutation may therefore be another alternative mechanism of WNT pathway activation in some colorectal SRCC. Besides, alterations of *DKK4*, *FZD10*, *AMER1*, and *AXIN2* may also contribute to β-catenin activation in SRCC, in the absence of *APC* and *RNF43* mutation [44]. In all, carcinogenesis driven by WNT pathway appear to be present in SRCC, often through mechanisms apart from *APC* mutation. The related molecular mechanisms need to be further investigated.

## RAS/RAF/MAPK Signaling Pathway

RAS/RAF/MAPK signaling is another important pathway in the colorectal adenoma-carcinoma sequence. Activating mutations in oncogene *KRAS* lead to a constitutively activated kinase cascade, resulting in EGFR-independent activation of the mitogen-activated protein kinase (MAPK) pathway and uncontrolled cell proliferation [46,53]. Oncogene *BRAF* is another component of RAS/RAF/MAPK pathway. Activating mutation of *BRAF* also results in signaling of this pathway [54,55]. In general, *KRAS* mutations occur in 30–40% of all CRC while *BRAF* mutations occur in 5–15% [54–58]. Signaling through this same pathway, mutations in *KRAS* and *BRAF* are both associated with poor prognosis in CRC and resistance to the treatment with EGFR inhibitors [53,54]. In addition, mutations of the two genes are mutually exclusive [55,56].

It has been recognized that most CRC containing *BRAF* (mostly *BRAF* V600E) have a CpG island methylator phenotype (CIMP), which is characterized by aberrant promotor methylation of many genes. By mediating the *MLH1* promotor methylation and epigenetic silencing this mismatch repair (MMR) gene, *BRAF* mutation has a strong correlation with MSI-H phenotype in sporadic CRC [55–57].

In a pathway analysis including 29 colorectal SRCC, mutation rates of several cancer driver genes were dramatically different between different histology subtypes. In SRCC, the mutation load in MAPK pathway was significantly lower than that of AC (20.7% vs. 60.5%, respectively) [44].

Many studies have shown that compared to AC, *KRAS* mutation is less common in colorectal SRCC [20,39,44,45,58,59]. Whereas some other studies revealed that there is no significant difference in the frequency of *KRAS* mutation between SRCC and AC [28,60].

As for the frequency of *BRAF* alteration in colorectal SRCC, previous studies also reported discrepant results. Many studies have reported significantly higher frequency of *BRAF* mutation in SRCC compared to AC. In addition, they invariably demonstrated a significant correlation between *BRAF* mutation and CIMP positive status and reported a relatively high incidence of MSI-H phenotype (24–48%) in colorectal SRCC [28,60–62]. However, some other studies have presented the opposite results. In a study including thirty-three patients with metastatic colorectal SRCC, *BRAF* mutation was observed in 8.6% cases and MSI-H status was found in 12.1% cases, which were comparable to those of AC [20]. In a Chinese cohort involving 61 cases of colorectal SRCC, *BRAF* mutation was identified in only 3.7% cases [58]. In a study conducting a comprehensive analysis of five colorectal SRCC using whole-exome and RNA sequencing analysis, none of the cases showed *BRAF* mutation and the authors attributed it to the microsatellite stable (MSS) status of all those cases [45]. Similarly, in a study analyzing only MSS/sporadic CRC, none of the eight SRCC demonstrated *BRAF* mutation [18]. In the comprehensive molecular pathology research of Alvi et al., features including somatic mutations, DNA methylation, MSI status, and biomarkers reflecting the tumor immune microenvironment (TIME) were analyzed in 44 cases of colorectal SRCC [62]. They found significant correlations between the hypermethylated genotype and CIMP positive, MSI-H, *BRAF* V600E mutation, female gender, advanced age, and proximal tumor location. Accordingly, they proposed that SRCC comprises two distinct genotypes, an MSI^+^/CIMP^+^/*BRAF* ^+^/CD3^+^/PD-L1^+^ hypermethylated genotype predominantly in the proximal colon and a hypomethylated genotype mostly in the distal colon, among which the hypermethylated subgroup may benefit from the immune checkpoint inhibitor (ICI) therapy [62]. As shown in Table 2, there are divergent results in the molecular characteristics of colorectal SRCC among different studies. We speculate that these differences may be largely due to the different proportions of the two methylation subtypes in different cohorts.

## *PIK3CA* and PI3K/AKT/mTOR Signaling Pathway

The phosphatidylinositol 3-kinase (PI3K)/protein kinase B (AKT)/mammalian target of rapamycin (mTOR) signaling pathway plays an important role in carcinogenesis, which is abnormally activated in various types of cancer. Phosphatidylinositol-4,5-bisphosphonate 3-kinase, catalytic subunit alpha polypeptide (*PIK3CA*), a gene encodes the p110α catalytic subunit of PI3K, plays an important role in the PI3K/AKT/mTOR pathway. Mutations in *PIK3CA* are present in approximately 15–20% of CRC, making it one of the major driver oncogenes in CRC [63,64]. In addition, *PIK3CA* mutations commonly coexist with KRAS mutations and lead to additive activation of the PI3K pathway [65]. In colorectal SRCC, lower frequency [59,61] or even absence [20,44,45] of *PIK3CA* mutation were reported. Li et al. [44], performed whole exome sequencing (WES) on 29 cases of SRCC and found that mutation of PI3K pathways is lower in SRCC compared to AC (6.9% vs. 46.1%). Mutations in *PIK3CA*, *PTEN* and *IRS2* were almost absent in SRCC. In addition, studies performing WES and gene panel sequencing analyses revealed that most driver genes associated with CRC, including *APC*, *KRAS*, and *PIK3CA*, are mutated at lower rates in SRCC [20,44,45].

## *SMAD4* and TGF-β Signaling Pathway

*SMAD4* is a tumor suppressor gene that regulates gene transcription and cell growth. It has been reported that CRC with loss of *SMAD4* expression is associated with aggressive tumor behavior, poor prognosis and chemoresistance to 5-fluorouracil-based therapy, as well as decreased tumoral and peritumoral immune infiltration [59,66]. In colorectal SRCC, frequent *SMAD4* alteration (20–30%) has been reported in several studies [20,44,45,59], which seems to be more common in comparison with AC (∼10%) [67]. Being a component of SMAD transcriptional complex, SMAD4 acts as a major regulator of the transforming growth factor-β (TGF-β) signaling pathway. In response to TGF-β, SMAD complex induces the epithelial-mesenchymal transition (EMT) process directly and indirectly. EMT, a process being characterized by destruction of epithelial cell junction and alteration of cell polarity, consequently generation of invasive cells with stem-cell like properties, is considered a key player in promoting tumor invasion and dissemination [45,68]. A study performing a gene set enrichment analysis (GSEA) revealed that SRCC-specific upregulated genes were enriched in the EMT and the “Stem Cell Up Regulation” processes [45]. Additionally, decreased E-cadherin expression and increased N-cadherin expression were observed in the tumor tissue of SRCC, which indicated the involvement of EMT process in tumorigenesis of SRCC [45]. SRCC with aberrant activated TGF-β/SMAD4 signaling pathway represents a refractory cancer subtype. Although there has been no medication targeting the loss of *SMAD4*, a few studies have shown the link between *SMAD4* loss and response to specific chemotherapeutic drugs such as topoisomerase inhibitors [69]. In addition, agents targeting the TGF-β/SMAD4 pathway may be another potential therapy [70].

## Microsatellite Instability Status, CpG Island Methylator Phenotype, and Chromosomal Instability

It has been recapitulated that ∼15% of CRC are MSI-H, which include ∼3% of inherited cancer susceptibility syndrome (predominantly Lynch syndrome) and ∼12% of sporadic CRC mainly due to silencing of the MMR genes (mostly by promotor methylation of *MLH1*) [56,57,71,72]. CRC with MSI-H is associated with advanced age, female gender, the proximal colon, poor differentiation, mucinous or SRC histology, *BRAF* mutation and CIMP positive status, as well as a better prognosis compared to CRC without MSI-H [72–74].

Despite small sample sizes, a number of studies have demonstrated that compared to AC, colorectal SRCC has a higher proportion of MSI-H phenotype, with a proportion of 19–48% according to literatures (Table 2) [28,60–62,75,76]. In contrast to the favorable prognostic value of MSI-H, the SRC histology is a well-accepted poor survival predictor. Most of the studies have shown that in colorectal SRCC, the MSI-H status does not appear to be a significant predictor of survival [27,75,76].

Kakar et al. [28] observed that different from the MSI-H AC, colorectal SRCC with MSI-H status has a significantly higher prevalence of chromosomal instability (CIN). This can be reflected by the fact that the loss of heterozygosity (LOH) presents in 80% of MSI-H SRCC and in only 14% of MSI-H AC. It was speculated that the aggressiveness of CIN phenotype outweighs any favorable effects of MSI-H in SRCC [28,75].

The CIMP is a distinct epigenotype which is characterized by widespread promoter methylation and silencing of tumor suppressor genes by methylation of their promoters. CIMP positive CRC commonly presents with MSI-H status due to methylation of *MLH1* and is associated with *BRAF* mutation [56,74]. Stratified with stage, improved cancer-specific survival (CSS) has been observed in CIMP positive CRC regardless of MSI status and *BRAF* mutation [74]. A number of studies have shown that CIMP positive status and *BRAF* mutation are more frequent in colorectal SRCC compared to AC [28,60,61,77]. According to the classification by methylation status [43,62], however, the CIMP positive SRCC seems to represent one of the two subtypes of colorectal SRCC, the hypermethylated genotype.

CIN is a gross genetic mutation that occurs at the chromosomal level, presenting as aneuploid or polyploid karyotype as well as multiple structural chromosomal changes such as translocations, allelic losses and amplifications [71,78]. CIN status strongly correlates with worse prognosis [71]. LOH is when one of a pair of alleles at a specific locus is missing, which is closely related to CIN, with LOH appearing frequently in CIN-high cases [78]. CRC with MSI-H tend to be diploid and CIN is not generally believed a major mechanism for the carcinogenesis of MSI-H CRC [28,71]. Being a common late event of colorectal carcinogenesis, 18q LOH was reported to be less frequent in SRCC (4/7, 57%) compared to AC (194/304, 64%) [60]. In contrast, Kakar et al. reported a higher LOH positive rate in SRCC than that in AC (93% vs. 70%, *p* = 0.04), with 18q LOH being found most frequent (6/17, 35.3%) in SRCC. Additionally, they did not observe significant difference in LOH between SRCC with MSI-H or MSS phenotype (80% vs. 100%, *p* = 0.3). The author argued that CIN, as manifested by LOH, is present in nearly all SRCC, including those with MSI-H [28].

Due to the limited number of publications, the related issues need further research to clarify.

## Tumor Immunology in Signet Ring Cell Carcinoma

It is increasingly recognized that the response of tumors to immunotherapies and most conventional anti-cancer therapies is associated with the immune contexture, which is determined by the density, composition, functional status and organization of immune cell infiltration within a tumor [79,80]. Introduction of checkpoint inhibitor (ICI) therapy provides remarkable achievements in multiple types of MSI-H or high tumor mutation burden(TMB-H) tumors. CRC patients with MSI-H have been well recognized as good candidates for ICI therapy [62,81]. In the study of Alvi et al. [62], adaptive immunity (CD3) and the immune checkpoint (PD-L1) were tested through immunohistochemistry (IHC) to evaluate the adaptive immune resistance in 44 cases of colorectal SRCC. In this cohort, colorectal SRCC was subclassified into hypermethylated (41%) and hypomethylated groups (59%) according to the DNA methylation status. The results showed that colorectal SRCC with MSI-H had a significantly higher infiltration of CD3^+^ T-lymphocytes and a higher PD-L1 expression compared to the MSS tumors. Similar trends were also observed in the MSI^+^/CIMP^+^/*BRAF* ^+^/CD3^+^/PD-L1^+^ hypermethylated group compared to the hypomethylated group. They concluded that the hypermethylated genotype of colorectal SRCC is an ideal candidate for the ICI therapy [62].

## Signet Ring Cell Carcinoma in Rectum

Epidemiological data show that rectal cancer accounts for 27–29% of all CRC in general [5,6,16]. In terms of SRC histology, rectal SRCC accounts for 17–21% of all colorectal SRCC cases [5,6,16,30], which indicates that the proportion of SRCC in the rectum is relatively lower. Analysis of the SEER data reveals that despite a stable incidence of rectal cancer for all ages, the incidence in patients under 40 has quadrupled from 1980 to 2010, and cancers in this group are 3.6 times more likely to have SRC histology [9]. These data indicate that incidence of rectal SRCC is increasing during the past decades, particularly in younger population. Unlike colon SRCC which occurs with approximately equal incidence in men and women, rectal SRCC is more common in men, with a male-to-female ratio of 1.6–2.1:1 [6,7,22]. Studies have shown that rectal SRCC is more aggressive, showing significantly worse survival, compared to colon SRCC [5,14,16]. A study based on the SEER database showed that rectal SRCC had significantly lower 5-year relative survival (5-y RS) rates compared to colon SRCC (21.1% vs. 28.6%) [16]. Another nationwide population-based study of Netherlands Cancer Registry (NCR) achieved quite similar results, showing the 5-y RS of 19.5% and 30.8% for rectal and colon SRCC, respectively [5]. Studies have shown that similar to the conventional CRC, SRCC in the right-sided (proximal) colon and left-sided (distal) colorectum also have different clinicopathological and molecular characteristics. SRCC with MSI-H status was significantly more frequently observed in the proximal colon than the distal colorectum, as shown in Table 2 [27,62,75,76]. In addition, poorly differentiated adenocarcinoma/MAC/SRCC of the proximal colon was reported to have significantly better DFS than distal cancers [82]. According to Alvi et al.’s classification of colorectal SRCC [62], rectal SRCC, as a part of distal SRCC, seems to be more in line with the hypomethylated genotype. Besides, rectal SRCC may also have some other unique features. Being a distinctive entity with an increased incidence in the young population, rectal SRCC deserves further investigations.

## Carcinomas With Signet Ring Cell Component

SRCC is defined as the presence of more than 50% of SRCs in the tumor. AC containing SRC component of less than 50% represents a substantial subgroup, but there is currently no formal SRCC designation for it [20]. In the study of Korphaisarn et al., the frequencies of somatic gene mutations in AC with SRC component varied between those observed in SRCC and AC. The author attributed this to the mixture of different components during tissue selection process and strongly recommended to define the patient as either SRCC or AC with SRC component [20]. In some other studies, however, no significant difference was observed for the clinicopathological and genetic characteristics between AC with SRC component of less than 50% and those with ≥50% [58,60,77]. In terms of clinical outcomes, most studies concluded that the SRC histology is associated with a significant poor survival of CRC patients, regardless of the percentage of SRC component [15,20,27,58,61,77]. Pande et al. proposed that the SRC component seems to confer the predisposition of the widespread metastatic pattern, which may explain the consistent poor prognosis of AC with SRC component, regardless of the proportion of SRCs [15].

Colorectal SRCC with different amount of extracellular mucin has also been investigated. Hartman et al. [76] designated colorectal SRCC into mucin-poor (extracellular mucin ≤ 49%) and mucin-rich (extracellular mucin > 50%) subgroups. Their study showed that the mucin-poor SRCC was characterized by the high frequency of lympho-vascular invasion and perineural infiltration, and significantly reduced survival compared to the mucin-rich group. Additionally, MSI-H status was mostly found in the mucin-rich group. However, in a study including 72 cases of colorectal SRCC, no significant difference in the MSI status was observed between tumors with ≥70% of extracellular mucin and those with <70%. And the article did not mention why 70% was used as the cutoff value [75]. It seems that subgrouping of SRCC according to the amount of extracellular mucin and its related clinical significance are worthy of further study.

## Treatment

Due to the relatively higher stage at diagnosis, Patients with SRCC are more likely to receive multimodality treatment including chemotherapy and radiotherapy [5,6,8,83]. Despite poor outcomes, studies have shown that patients with colorectal SRCC can benefit from chemotherapy. In a large-scale study that included 1,972 SRCC patients, Hugen et. al [5]. found that adjuvant fluorouracil-based chemotherapy for stage III colon cancer yielded similar benefits for the conventional and SRC subtypes. A recent study including 1,675 patients with stage II/III colorectal SRCC showed that postoperative chemotherapy was an independent prognostic factor for better CSS and OS (CSS: HR = 0.719, 95% CI 0.612–0.844, *p* < 0.001); (OS: HR = 0.618, 95% CI 0.537–0.713, *p* < 0.001) [84].

Hugen et al. [5] proposed that chemotherapy benefits colorectal SRCC, the poor outcomes of patients may be related to the more advanced stage and the deviant metastatic pattern of SRCC. Peritoneal metastasis (PM), frequent in SRCC, represents a clinical difficulty that can hardly be treated with cure intent [5]. A population-based study revealed that compared to surgery, chemotherapy was associated with better survival for colorectal SRCC patients with PM [4]. Although with some benefits, the standard chemotherapeutic regimens for patients with PM are mostly palliative treatment that cannot cure the disease.

As for CRC with PM, it has been reported that cytoreductive surgery (CRS) with hyperthermic intraperitoneal chemotherapy (HIPEC) is beneficial for selected patients [85]. However, properly selecting candidates who may benefit from this treatment is very important. It was reported that improved survival was only observed when complete cytoreduction was achieved [86]. It has been shown that unfavorable factors in tumor biology, such as *RAS*/*RAF* mutations, high-grade tumors, and SRC histology, are associated with worse survival in patients receiving CRS/HIPEC [87]. A number of prognostic scoring systems have been developed for optimal clinical practice, but no ideal scoring system has been recognized. Related issues are still being improved.

Studies have revealed that colorectal SRCC is a heterogeneous subgroup with different underlying molecular mechanisms. It is therefore important to perform individualized therapy according to the molecular characteristics of different patients. For instance, for tumors with loss of *SMAD4* expression, regimens with topoisomerase inhibitors may be recommended. For SRCC with the MSI^+^/CIMP^+^/*BRAF* ^+^/CD3^+^/PD-L1^+^ hypermethylated genotype, ICI therapy may be a potentially effective treatment [62]. In addition, systemic treatment with combined irinotecan (CPT-11)/panitumumab regimen was reported to be effective in an end-staged colon SRCC patient with disseminated carcinomatosis [34]. New medication targeting the specific signaling pathway or multiple pathways represents the future research direction.

Due to the special metastatic pattern of SRCC, which includes diffuse spread of small lesions and lower incidence of liver/pulmonary metastasis, the follow-up plan for colorectal SRCC should also be different from that for AC. Physicians cannot rely solely on imaging studies including computed tomography (CT) scan or magnetic resonance imaging (MRI). It is recommended to pay more attention to the monitoring of tumor markers including cancer embryonic antigen (CEA) and carbohydrate antigen 19-9 (CA19-9). PET-CT can be performed at an earlier stage. In these patient groups, early detection of peritoneal metastases should be priority [15,32]. Besides, novel molecular markers such as circulating tumor DNA (ctDNA) can be used as promising monitoring indicators [88,89].

## Conclusion

As a rare but aggressive subtype of CRC, SRCC has distinct clinicopathological and molecular characteristics. Due to the unfavorable clinical features including advanced tumor stage at diagnosis, high tumor grade, high rate of lymph node involvement, early and diffuse distant metastasis, SRCC is associated with a bleak prognosis. In aspects of molecular biology, colorectal SRCC presents lower mutation burden in the canonical WNT, MAPK, and PI3K pathways, with most driver genes in conventional CRC (*APC*, *KRAS*, and *PIK3CA*, etc.) being mutated at lower rates in SRCC. In contrast, some SRCC-specific altered genes and signaling pathways were reported to be implicated in the “EMT” and “Stem Cell Up Regulation” processes (such as *RNF43*, *CDH1*, *SMAD4*, β-catenin and TGF-β pathway), which were believed the reason of the aggressive tumor biology and the chemoresistance of SRCC. High frequency of *BRAF* mutation was observed in many studies of colorectal SRCC while it was not always the case. It was proposed that SRCC comprises two distinct genotypes, an MSI^+^/CIMP^+^/*BRAF* ^+^/CD3^+^/PD-L1^+^ hypermethylated genotype predominantly in the proximal colon and a hypomethylated genotype mostly in the distal colon, in which the hypermethylated subgroup is a potential candidate of the ICI treatment. The different proportions of the two methylation subtypes may explain the discrepancy in *BRAF* mutation rates in different studies. For such a refractory disease, some new medication targeting the specific pathways of SRCC have been attempted. However, more in-depth researches are still needed to gain further understanding, in order to achieve substantial improvements in the treatment of this challenging disease.
